# Repeated dosing of myrrh, chamomile extract, and coffee charcoal reveals potential health-beneficial effects in patients with irritable bowel syndrome in the M-SHIME simulator

**DOI:** 10.1371/journal.pone.0348791

**Published:** 2026-05-27

**Authors:** Meinolf Wonnemann, Bartosz Lipowicz, Cindy Duysburgh, Chloë Rotsaert, Lynn Verstrepen, Massimo Marzorati, Jost Langhorst

**Affiliations:** 1 Repha GmbH Biologische Arzneimittel, Langenhagen, Germany; 2 ProDigest, Zwijnaarde, Belgium, Germany; 3 Center for Microbial Ecology and Technology (CMET), Faculty of Bioscience Engineering, University of Ghent, Gent, BelgiumGermany; 4 Department for Integrative Medicine, Medical Faculty, University of Duisburg-Essen, Essen, Germany; 5 Department of Internal and Integrative Medicine, Sozialstiftung Bamberg, Bamberg, Germany; Hong Kong Baptist University, HONG KONG

## Abstract

**Objective:**

To define the potential functional roles of a herbal preparation of myrrh, chamomile extract, and coffee charcoal in patients suffering from diarrhea dominant irritable bowel syndrome (IBS-D).

**Methods:**

The study utilized the Mucosal Simulator of the Human Intestinal Microbial Environment (M-SHIME^®^) with proximal (PC) and distal colon (DC) compartments and fecal samples from four IBS-D donors. Eight-day (d) repeated dosing with the herbal product (6 tablets/day) was initiated compared to a negative control. Changes in microbial metabolism and community composition were assessed, and colonic ferments were evaluated for their effects on intestinal barrier permeability and cytokine production in a Caco-2/THP-1 co-culture model.

**Results:**

Product treatment significantly increased gas pressure versus negative control, indicating microbial fermentative activity. Product supplementation significantly increased proximal acetate (d3, d5), propionate (d3), and butyrate (d5, d8) levels (p < 0.05 for all), while no significant changes were observed distally. Ammonium levels were significantly elevated following product supplementation in PC (d3, d5, d8; p < 0.05) and DC (d8; p < 0.01), though remained within physiological range. Repeated dosing enriched members of *Bifidobacteriaceae*, Bacteroidota, *Lachnospiraceae*, and *Butyricicoccus* versus negative control. Treated colonic ferments had a protective effect on intestinal membrane integrity (DC; p < 0.001) and positive immunomodulatory effects (increased IL-10, PC and DC [both p < 0.0001], and IL-6, PC [p < 0.001] and DC [p < 0.01]) in Caco-2/THP-1 co-cultures.

**Conclusions:**

Treatment with a herbal preparation of myrrh, chamomile extract, and coffee charcoal showed a potential beneficial effect on the microbiota of patients with IBS-D *in vitro*, suggesting further exploration of its efficacy in IBS-D and other chronic gastrointestinal disorders.

## Introduction

Irritable bowel syndrome (IBS) is a chronic, functional gastrointestinal disorder that causes abnormalities in bowel function and abdominal pain [1]. It is associated with a high burden of disease and decreased quality of life [2]. The two main bowel subtypes are IBS with diarrhea (IBS-D) and IBS with constipation (IBS-C). Non-pharmacological interventions include exercise, stress reduction, and dietary changes [1]. The American Gastroenterological Association Guideline recommends tricyclic antidepressants and antispasmodics for patients with IBS of any subtype, and suggests that eluxadoline (mixed mu- and kappa-opioid receptor agonist/delta-opioid receptor antagonist), loperamide (anti-diarrheal), rifaximin (antibiotic), and alosetron (5-HT3 receptor antagonist) may be considered for treating patients with IBS-D [2]. There is also interest in phytotherapeutic options, such as peppermint oil, for aiding in treatment [3].

The combined herbal preparation of myrrh, chamomile extract, and coffee charcoal (Myrrhinil-Intest^®^; Repha GmbH, Langenhagen, Germany) has been used to treat diarrhea for well over 50 years [4]. More recently, there has been interest in its effectiveness as a complementary herbal treatment for chronic gastrointestinal disorders. To address this, a clinical study was conducted in patients with ulcerative colitis who were in remission [5]. The herbal preparation was well tolerated and effective as maintenance therapy. Exploratory analyses reported a significant reduction in short-chain fatty acid (SCFA) levels during flares in patients treated with the comparator (mesalamine) but not in patients treated with the herbal preparation [6]. Patients treated with the herbal preparation appeared to have an increase in CD4 + CD25 high regulatory T cells during acute flares that was not observed with mesalamine [7]. These data suggest distinct mechanisms of action for the two treatments.

The herbal preparation and its individual components have been tested *in vitro*, demonstrating a stabilizing effect on the intestinal barrier and anti-inflammatory properties in experiments with lipopolysaccharide (LPS)-activated THP-1 cells [8–11]. Antispasmodic effects have been observed for myrrh, chamomile flower extract, and the complete herbal formulation, which may be beneficial to patients with IBS-D or other chronic gastrointestinal disorders [12,13]. Additionally, the herbal formulation is reported to have anti-fungal effects [14–15].

Patients with IBS-D have increased intestinal permeability [16,17], which has been associated with decreased expression and an altered subcellular distribution pattern of the tight junction proteins zonula occludens (ZO)-1 and occludin [18,19]. Increased serum tumor necrosis factor (TNF)-α, interleukin (IL)-1β, and IL-6 levels in patients with IBS-D suggest that inflammation is involved in the disorder [20]. Recent views on IBS suggest overlap with other functional gastrointestinal diseases, including ulcerative colitis [1]. Thus, prior reports of improved intestinal barrier function and immunomodulatory effects with the herbal preparation, and of its effectiveness in patients with ulcerative colitis suggest that treatment may benefit patients with IBS-D. Furthermore, as the gut microbiome has been increasingly linked with health and disease during the past decades, assessment of the effect of the herbal preparation on intestinal metabolic activity and community composition could provide additional evidence on the potential mode-of-action of the product in the area of gastrointestinal diseases. The Mucosal Simulator of the Human Intestinal Microbial Environment (M-SHIME^®^) is a validated dynamic *in vitro* gut model where endogenous colonic microbiota are cultured under conditions representative for the luminal and mucosal intestinal environment [21–23]. M-SHIME^®^ was used to explore the effects of repeated administration of the herbal preparation on the gut microbiota of patients with IBS-D, and to define its potential functional roles in relation to human health, which have not been shown before for the product of interest. Specifically, changes in microbial metabolite production and microbial community composition were assessed, as were the effects of colonic ferments on intestinal barrier permeability and their immunomodulatory properties.

## Materials and methods

### Herbal test product

The investigational test product was Myrrhinil-Intest^®^ (batch 22A0817; Repha GmbH), containing 100 mg myrrh, 70 mg chamomile extract, and 50 mg coffee charcoal as active ingredients. It is registered as a traditional medicinal product and provided as a coated immediate release tablet formulation. During the current study, the product was administered at an *in vitro* test dose of six tablets per day, equally spread over the three feeding cycles, corresponding to the *in vivo* dosing strategy of four tablets three times a day. Tablets were crushed prior to addition to the stomach compartment together with the nutritional medium. As the herbal test product contained high levels of sucrose (18.1%), which is not considered as part of the active ingredients of Myrrhinil-Intest^®^, an equal amount of sucrose was added to the negative control during the current experiment to be able to assess the effect of the active ingredients of Myrrhinil-Intest^®^ only. Therefore, a dose of 596.8 mg sucrose per day was administered to the negative control arm, equally spread over the three feeding cycles.

### *In vitro* simulation

Stool samples were collected from four adults (donors A-D) with diagnosed IBS-D, with recruitment taking place in the period 01 May – 31August 2023. Individuals were eligible to donate if they were aged between 18 and 50 years, were clinically diagnosed with IBS-D, and had not taken any antibiotics within the four months prior to sample donation. Fecal samples were processed and stored at –80°C (see S1 Text). Fecal materials were collected and used as approved by the Ethics Committee of the University Hospital Ghent (reference number ONZ-2022–0267; approved on 29 July 2022).

This study employed the M-SHIME^®^ (ProDigest and Ghent University, Ghent, Belgium) which simulates both the mucus-associated and luminal colonic microbial environments, with details of the full setup being published previously [23,24]. The configuration used for this study included two colon regions per test condition, i.e., the proximal colon (PC; pH 5.75–5.95; 20h retention time; 500 mL volume) and the distal colon (DC; pH 6.6–6.9; 32h retention time, 800 mL volume). Mucin-coated beads were added to both the PC and DC compartments to simulate the mucosal environment [25].

In the current study, eight sets of the M-SHIME^®^ configuration were run enabling evaluation of the herbal medicinal test product versus a negative control for four different donors in a repeated-dosing design (S1 Fig). At the start of the experiment, the PC and DC reactors were inoculated with a 5% (v/v) cryopreserved fecal inoculum containing 20% (w/v) fecal material from donor A, donor B, donor C, or donor D and the microbial community was allowed to stabilize for two days following overnight colonization and growth. Subsequently, the reactors were supplemented three times daily with the herbal test product or negative control for 8 days (d1 through d8). Throughout the study, the reactors were fed with basic M-SHIME^®^ nutritional medium (14.6 g/L PDNM002B, ProDigest, Belgium) and pancreatic juice (12.5 g NaHCO_3_, 6 g/L oxgall, and 0.9 g/L pancreatin) three times daily.

### Analysis of metabolic activity

Samples were collected at d1, d3, d5, and d8. SCFA (acetate, propionate, and butyrate) and branched chain fatty acids (BCFA; sum of isobutyrate, isovalerate and isocaproate) were isolated using liquid-liquid extraction and their concentrations were determined using capillary gas chromatography, coupled with a flame ionization detector [26]. Lactate concentrations were determined using an Enzytec™ kit (R-Biopharm, Darmstadt, Germany) according to the manufacturer’s instructions. Ammonium was measured as ammonium-nitrogen (NH_4_^+^-N) and quantified using the indophenol blue spectrophotometric method according to Tzollas et al. [27]. Each measurement was performed in a single repetition.

### Gas production

Given the restrictions and difficulties associated with measuring gas pressure in continuous models, short-term colonic simulations were used to assess gas production [28]. PC samples collected at d1 (i.e., prior to administration of the first treatment) and d8 (i.e., at the end of the treatment phase) were used as the inoculum. At the start of the short-term colonic simulation, the herbal test product or control (at a similar dose as used in the M-SHIME^®^ experiment) and 10% (v/v) inoculum were added to colonic nutritional medium (PD01; ProDigest) and incubated under anaerobic conditions for 48 h at 37°C with mild (90 rpm) shaking. A hand-held pressure indicator (CPH6200; Wika, Echt, The Netherlands) was used to measure changes in gas pressure between 0 h, 3 h, 6 h, 24 h, and 48 h.

### Analysis of microbial community composition

Samples were collected on d1, d3, d5, and d8. DNA was isolated using the method described by Duysburgh et al. [29]. 16S-targeted sequencing, read assembly, and cleanup were conducted as previously described (S2 Text) [29].

To convert the metagenomics data from relative abundances to absolute abundances, the total number of bacterial cells was quantified for each luminal sample using flow cytometry (BD Accuri C6 Plus Flow Cytometer [BD Biosciences, Franklin Lakes, NJ, USA]; high flow rate). A SYTO channel threshold of 700 was used to distinguish bacterial cells from signal noise and medium debris. Absolute abundances were calculated by multiplying the relative abundance by the total cell count [30].

### Caco-2/THP-1 co-culture model

Caco-2 cells (HTB-37; American Type Culture Collection) and phorbol-12-myristate-13-acetate differentiated THP-1-Blue™ cells (InvivoGen; San Diego, California, USA) were used in the co-culture model as previously described [31,32]. Colonic suspensions were collected on d8, sterile filtered (0.22 µM), diluted 1:5, then added to the co-cultures (24 h at 37˚C with 5% CO_2_ in a humidified atmosphere). Transepithlial electrical resistance (TEER) was measured at baseline and 24 h. After discarding the basolateral medium, the cells were stimulated with 500 ng/mL ultrapure LPS (*Escherichia coli* K12, InvivoGen) for 6 h (37˚C, 5% CO_2_, humidified atmosphere). Basolateral supernatants were collected and cytokine levels (including anti-inflammatory cytokines IL-10 and IL-6 [the latter considered anti-inflammatory in the used *in vitro* model, as it is crucial for epithelial regeneration and wound healing, with butyrate being able to increase its expression [33]],and pro-inflammatory cytokines TNF-α and IL-1β) were quantified using a Luminex^®^ multiplex (ThermoFisher Scientific, Waltham, Massachusetts, USA) per the manufacturer’s instructions. Samples from the colonic incubations were used in the cell co-culture assay as technical triplicates.

### Statistical analysis

Results from gas pressure, acetate, propionate, butyrate, lactate, BCFA, ammonium, TEER, and cytokine assays were compared between the treatment and negative control groups using unpaired Student’s t-tests with single values as input. A p-value <0.05 was considered statistically significant. All statistical analyses were performed using GraphPad Prism version 10.5.0 for Windows (GraphPad Software, San Diego, CA, USA).

Hierarchical clustering of Euclidean distances between samples using Ward’s minimum variance method was used to conduct beta-diversity analysis. Adegenet v2.1.10 was used to construct a Discriminant Analysis of Principal Components (DAPC) plot with two discriminants and 80% percent of retained variance in the principal components [34,35]. Both analyses were run in R v4.3.1 and were plotted using ggplot2 (v3.4.2).

Relative abundance data were obtained by sum scaling and then subjected to Linear Discriminant Effect Size (LEfSe) analysis [36]. Features meeting p ≤ 0.05 for the Kruskal-Wallis and Wilcoxon tests are shown on the LEfSe table. No minimal score restrictions were used for the linear discriminant analysis (LDA). A score of ≥2 is generally considered biologically relevant. TreeclimbR analysis was also conducted [37]. Bacterial enrichments with a -log(p-value) > 1.3 were considered statistically significant. Taxa were classified using four categories: (1) not significant and not biologically relevant (−2 < log_2_ fold change [FC] <+2, and -log_10_[p-value] < 1.3); (2) biologically relevant, but not statistically significant (log_2_FC < −2 or log_2_FC > +2, and -log_10_[p-value] < 1.3); (3) statistically significant, but not biologically relevant (−2 < log_2_FC < +2, and -log_10_[p-value] > 1.3); and (4) biologically and statistically significant (log_2_FC < −2 or log_2_FC > +2, and -log_10_[p-value] > 1.3). The R package stats4 (The R Foundation for Statistical Computing, Vienna, Austria) was used for LEfSe pairwise comparisons, and the R package MASS v7.3.58-3 was used to calculate LDA scores between the test product and negative control taxon abundances. TreeclimbR analysis was run using treeclimbR v0.1.5 and edgeR v3.42.421. Benjamini-Hochberg multiple testing correction was used, and the alpha-level was set at 0.05. Both analyses were run in R v4.3.1.

## Results

### Metabolic activity

#### Gas pressure.

**Fig 1:**
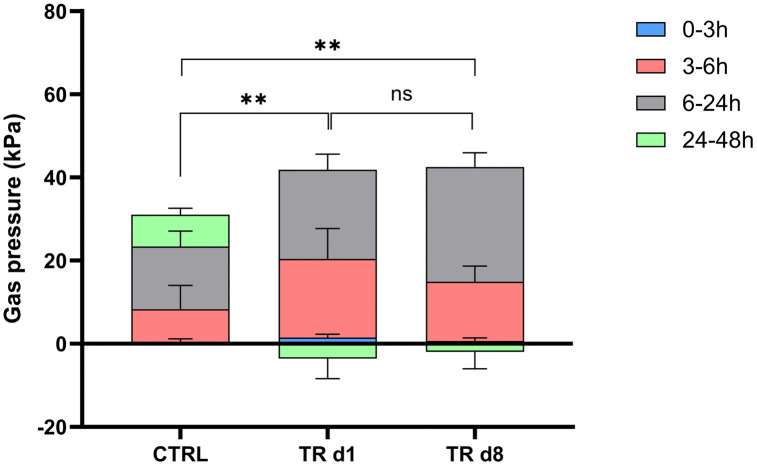
Gas pressure. Changes in gas pressure (kPa) between the negative control and treatment with the herbal test product (myrrh, chamomile extract, and coffee charcoal) at d1 and d8 in M-SHIME^®^ colonic incubations at different time intervals (0-3h, 3-6h, 6-24h, and 24-48h following the start of the colonic incubations). Results are presented as mean ± standard deviation across four donors with three measurements per donor (n = 12). Unpaired Student’s t-tests were used to compare changes observed between the different test conditions. **p < 0.01. CTRL, negative control; d, day; M-SHIME^®^, Mucosal Simulator of the Human Intestinal Microbial Environment; ns, not significant; TR, treatment.

Gas pressure was significantly increased between the control and test product treatment, following both the acute first dosing (d1) and repeated product administration at d8 (p < 0.01 for both). Gas pressure was not significantly different between d1 and d8 of test product treatment (Fig 1).

#### Metabolites.

**Fig 2:**
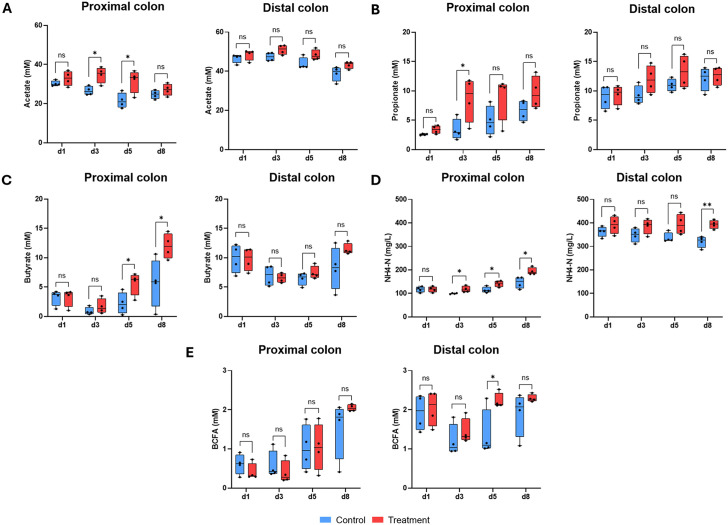
Metabolic activity. Box plots showing changes in acetate (mM) **(A)**, propionate (mM) **(B)**, butyrate (mM) **(C)**, ammonium (mg/L) **(D)**, and BCFA (mM) **(E)** levels between the negative control and treatment with the test product (myrrh, chamomile extract, and coffee charcoal) in the proximal and distal colon compartments across the treatment period (d1, d3, d5, d8) in M-SHIME^®^ colonic incubations. Results are presented in a box plot displaying the value for each of the four donors with a dot. The horizontal line represents the median across donors. Unpaired Student’s t-tests were used to compare changes observed for treatment with the test product versus the negative control. *p < 0.05. **p < 0.01. BCFA, branched chain fatty acid; d, day; M-SHIME^®^, Mucosal Simulator of the Human Intestinal Microbial Environment; NH4-N, ammonium; ns, not significant.

In the PC, acetate levels across donors were significantly increased with the treatment versus control on d3 and d5, but levels were similar on d1 and d8 (Fig 2A). Propionate levels tended to increase over time and were similar between treatment and control at all timepoints except d3, where propionate levels were significantly higher following treatment (p < 0.05) (Fig 2B). Butyrate production was not significantly different between the treatment and control groups at d1 and d3, but was significantly increased following product administration versus control on d5 and d8 (p < 0.05 for both) (Fig 2C). In the DC, there were no significant differences between treatment and control for acetate, propionate, or butyrate at any timepoint (Figs 2A-C). Lactate levels were high in the PC at the start of the experiment (>5 mM for some donors), indicating a lack of established cross-feeding interactions within the microbial community and confirming microbiome dysbiosis in the IBS-D fecal samples. While lactate levels decreased over time, negligible differences in lactate concentrations were detected between the treatment and control (S2 Fig).

Ammonium levels tended to increase slightly over time and were significantly higher with treatment versus control at d3, d5, and d8 (all p < 0.05) in the PC and at d8 (p < 0.01) in the DC (Fig 2D). Levels of BCFA were similar between treatment and control in both the PC and DC at all timepoints, except at d5 in the DC compartment where BCFA levels were significantly higher following product administration (p < 0.05) (Fig 2E).

### Microbial community composition

#### Beta-diversity.

Beta diversity analysis demonstrated that the treatment effects did not surpass interindividual variability, as clustering was only observed among the different donors and not according to treatment versus control (S3 Fig).

#### Differential abundance analysis.

**Fig 3:**
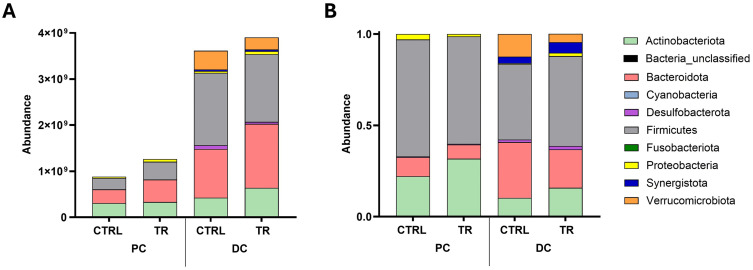
Microbial community composition at phylum level. Stacked bar plots showing absolute phyla abundances (cells/mL) in the lumen **(A)** and relative phyla abundances in the mucosal compartment **(B)** on day 8 following treatment with the test product (myrrh, chamomile extract, and coffee charcoal) versus the negative control. Results are presented as average across donors (n = 4). Flow cytometry was used to determine the total number of bacterial cells in the luminal samples. CTRL, negative control, DC, distal colon; PC, proximal colon; TR, treatment.

In the luminal environment, the absolute abundance at d8 (timepoint with maximal effect) was higher following treatment versus control in the PC and DC (Fig 3A). The distribution of phyla was similar between treatment and control in both colon compartments. The main phyla in the PC were Actinobacteriota, Bacteroidota, Firmicutes, and Proteobacteria. In the DC, the main phyla were Firmicutes, Bacteroidota, Actinobacteriota, and Verrucomicrobiota. In the mucosal environment, some differences in phyla relative abundances were observed between treatment and control (Fig 3B). In the PC, the relative abundance of the Actinobacteriota phylum was enhanced following treatment versus control, while the opposite was true for the remaining predominant phyla (Bacteroidota, Firmicutes, and Proteobacteria). In the DC, the Actinobacteriota, Firmicutes, and Synergistota phyla had an increased relative abundance with the treatment versus control, with the opposite being true for Bacteroidota and Verrucomicrobiota, the other predominant phyla.

Bacterial enrichments at d8 at the genus level are shown in Table 1. In both the luminal PC and DC, members of the *Veillonellaceae* family were biologically and statistically enriched following product administration versus control. In the luminal PC *Prevotella_9* spp were biologically enriched and the *Parabacteroides* genus was biologically and statistically enhanced with treatment versus control. Both are members of the Bacteroidota phylum, which contains numerous primary substrate degraders that produce acetate and/or propionate [39]. Members of the Firmicutes phylum were also enriched with treatment, including *Butyricicoccus* (statistically enriched). Additionally, *Escherichia-Shigella* was biologically and significantly enriched following supplementation with treatment. In the luminal DC, members of the Bacteroidota phylum, *Bacteroidales_unclassified* and *Prevotellaceae_ge*, were statistically and biologically enriched, respectively, with treatment versus control. Several members of the *Lachnospiraceae* family, linked with butyrate and acetate production, were both biologically and statistically enriched, and members of the *Ruminococcaceae* family, linked with butyrate production, were biologically enriched following treatment. In both the mucosal PC and DC, members of the Bacteroidota phylum were enriched with treatment versus control (*Bacteroidales_unclassified* [statistically enriched; PC only], *Prevotellaceae_ge* [biologically enriched; PC only], *Parabacteroides* [biologically and statistically enriched; PC and DC]). In the mucosal PC, members of the *Bifidobacteraceae* family were statistically enriched with treatment versus control, as was *Phascolarctobacterium* (biologically enriched) and members of the *Lachnospiraceae* family (statistically enriched). *Burkholderiales_unclassified* was statistically enriched following treatment. In the mucosal DC, *Gordonibacter* was statistically enriched and *UBA1819* (*Ruminococcaceae* family) was biologically and statistically enhanced. Interestingly, the *Veillonella* genus was biologically and statistically reduced in both the luminal and mucosal environments of both the PC and DC compartments.

**Table 1:** Effects on microbial community composition following product supplementation.

			Lumen		Mucus	
**Phylum**	**Family**	**Genus**	**PC**	**DC**	**PC**	**DC**
Actinobacteriota	*Bifidobacteriaceae*	*Bifidobacteriaceae_unclassified*			+	
	*Coriobacteriaceae*	*Coriobacteriaceae_unclassified*			–	
	*Eggerthellaceae*	*Gordonibacter*				+
Bacteroidota	*Bacteroidales_unclassified*	*Bacteroidales_unclassified*		+	+	
	*Prevotellaceae*	*Prevotella_9*	+			
		*Prevotellaceae_ge*		+	+	
	*Rikenellaceae*	*Alistipes*		–		
	*Tannerellaceae*	*Parabacteroides*	+		+	+
Firmicutes	*Acidaminococcaceae*	*Phascolarctobacterium*			+	
	*Bacilli_unclassified*	*Bacilli_unclassified*			–	
	*Butyricicoccaceae*	*Butyricicoccus*	+			
	*Clostridiaceae*	*Clostridium_sensu_stricto_1*		+		+
		*Clostridiaceae_unclassified*				+
	*Lachnospiraceae*	*Anaerostipes*			+	
		*Blautia*		+		
		*Fusicatenibacter*			+	
		*Lachnoclostridium*		–		
		*Lachnospiraceae_ge*			+	
		*Lachnospiraceae_ND3007_group*		+		
		*Lachnospiraceae_unclassified*		+		
		*Roseburia*		–		–
	*Lactobacillaceae*	*Lactobacillaceae_ge*	–	–		
		*Limosilactobacillus*			–	
	*Oscillospiraceae*	*Flavonifractor*			+	
	*Planococcaceae*	*Lysinibacillus*	–			
	*Ruminococcaceae*	*Faecalibacterium*		+		
		*UBA1819*		+		+
	*Veillonellaceae*	*Anaeroglobus*	+		+	
		*Dialister*	+			
		*Veillonella*	–	–	–	–
		*Veillonellaceae_unclassified*	+	+	+	
	*Veillonellales- Selenomonadales_unclassified*	*Veillonellales- Selenomonadales_unclassified*			–	
Proteobacteria	*Burkholderiales_unclassified*	*Burkholderiales_unclassified*			+	
	*Enterobacteriaceae*	*Escherichia-Shigella*	+			
		*Klebsiella*			–	
	*Sutterellaceae*	*Sutterella*				–
Verrucomicrobiota	*Akkermansiaceae*	*Akkermansia*				–

Overview of supplementation-induced enrichments (+) or reductions (-) compared with the negative control in the luminal and mucosal environments of the PC and DC compartments at d8. Pink shading indicates statistically and biologically enriched or reduced (p < 0.05, FC > 4), blue shading indicates statistically enriched or reduced (p < 0.05, FC < 4), green shading indicates biologically enriched or reduced (p > 0.05, FC > 4). DC, distal colon; FC, fold change; ge, genus; PC, proximal colon.

### Caco-2/THP-1 co-culture

#### Epithelial barrier integrity.

**Fig 4:**
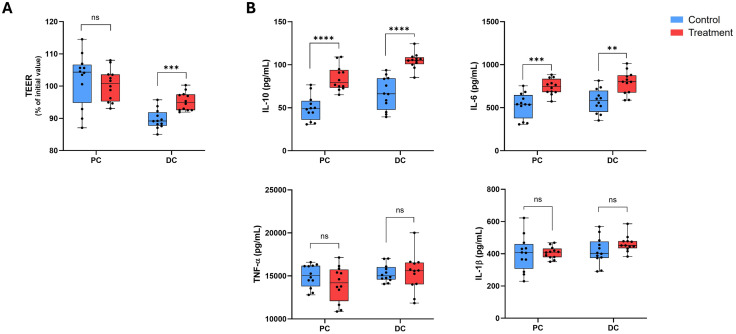
Cell culture responses. Box plots showing the effect of colonic suspensions (negative control and treatment, day 8) on TEER of the Caco-2/THP-1 co-cultures **(A)** and on cytokine levels **(B)**. TEER was measured 24 h after treatment of the co-cultures with colonic supernatants collected from the M-SHIME^®^ PC and DC compartments on day 8. Each 24 h value was normalized to its corresponding 0 h value and is shown as percentage of initial value. Cytokine levels were measured after 24 h exposure of the co-cultures to M-SHIME^®^ colonic supernatants collected on day 8 followed by 6 h stimulation with lipopolysaccharide. Results are presented in a box plot displaying the value for each of the replicates (four donors, three measurements per donor; n = 12) with a dot. The horizontal line represents the median across donors. Unpaired Student’s t-tests were used to compare changes observed for treatment with the test product versus the negative control. **p < 0.01. ***p < 0.001. ****p < 0.0001. DC, distal colon; IL, interleukin; M-SHIME^®^, Mucosal Simulator of the Human Intestinal Microbial Environment; ns, not significant; PC, proximal colon; TEER, transepithelial electrical resistance; TNF, tumor necrosis factor.

TEER data obtained using PC and DC supernatants collected at d8 are shown in Fig 4A. PC supernatants had little effect on membrane permeability, with no significant difference between treatment and control. Conversely, DC supernatants tended to reduce the TEER (versus the initial value), though supernatants from the treatment group minimized this reduction, demonstrating a significantly higher TEER (% of initial value) than the control (p < 0.001).

#### Cytokines.

Across donors, PC and DC ferments collected on d8 following treatment with the herbal test product significantly enhanced IL-10 production by LPS-activated THP-1 cells compared with control ferments (p < 0.0001 for both) (Fig 4B). A similar result was obtained for IL-6 (PC, p < 0.001; DC, p < 0.01) (Fig 4B). No significant differences were observed between the effect of treatment and control supernatants collected from the PC or DC on TNF-α or IL-1β levels (Fig 4B).

## Discussion

This study had three important results. First, using *in vitro* colonic simulations with fecal samples from patients with IBS-D, our data show that repeated administration with a herbal preparation of myrrh, chamomile extract, and coffee charcoal stimulated the microbial production of acetate, propionate, and butyrate, which are key to intestinal health. Second, proteolytic fermentation was enhanced, as seen by the increase in ammonium levels towards the end of the treatment period, though remaining within physiological range; microbial fermentation was further confirmed by increased gas production following product administration. Third, treated colonic ferments protected against inflammation-induced damage to the intestinal barrier and had immunomodulatory effects *in vitro*. These results build upon previous *in vitro* observations and findings from clinical trials in patients with ulcerative colitis, confirming beneficial properties and providing initial evidence that they can also be observed in the context of IBS-D. Importantly, bacterial phyla, families, and genera that are known to produce acetate, propionate, and butyrate were enriched, providing mechanistic evidence for the observed changes in microbial metabolism and the protective effects on intestinal epithelial cells.

In the PC compartment, treatment with the herbal test product resulted in significantly higher levels of acetate, propionate, and butyrate compared with the negative control at different timepoints. A clinical study in patients with ulcerative colitis comparing the effects of mesalamine and the same herbal test product (myrrh, chamomile extract, and coffee charcoal) showed that patients treated with mesalamine had a significant decrease in total SCFA and butyrate levels during a clinical flare (versus baseline values) [6]. In contrast, patients treated with the herbal preparation did not experience a significant decrease in total SCFA or butyrate levels, indicating that the herbal preparation supports SCFA production to some extent. This aligns with the present study findings of increased levels of SCFAs, including significant effects on butyrate following repeated dosing, with herbal treatment. To further investigate this finding mechanistically, the present study evaluated changes in the gut microbiota following product supplementation. Indeed, biologically and statistically significant enrichments in bacterial families and genera with SCFA-producing abilities were reported with treatment versus control. Specifically, *Bifidobacteriaceae* members (acetate and lactate) [38], Bacteroidota members (acetate and propionate) [39], and *Lachnospiraceae* members (butyrate and acetate) [40] were enriched in the mucosal PC environment following repeated product administration. Additionally, *Butyricicoccus*, a strong producer of butyrate [41,42], was enriched in the luminal PC environment. These data demonstrate that the herbal test product influences the composition of the gut microbiota, supporting the enrichment of bacteria that produce health-promoting SCFAs. This provides a potential mechanism for the increased SCFA production observed with treatment, which could be of interest in certain gastrointestinal disorders.

A further confirmation of enhanced fermentative activity in the colonic environment following product administration was linked to the observed increase in gas pressure. While the potential clinical relevance of this observation, particularly in the context of IBS‑D where increased gas production and gas handling abnormalities are associated with symptoms such as bloating, abdominal discomfort and altered bowel habits [43], is of importance, gas pressure remained below 60 kPa under all experimental conditions, which is within commonly reported physiological ranges for *in vitro* colonic fermentation systems and does not necessarily equate to excessive or pathological gas production [44]. Moreover, the absence of a significant difference in gas pressure between acute (d1) and repeated (d8) administration suggests that the intervention did not induce a progressive or cumulative increase in gas formation over time, which may indicate microbial metabolic adaptation rather than dysregulated fermentation. An additional observation was the significant increase in ammonium levels detected in the DC. Ammonium is a well-recognized by-product of colonic proteolysis, and sustained elevations have been associated with adverse effects on colonic epithelial function, including impaired barrier integrity, altered epithelial metabolism, and potential pro-inflammatory signalling [45]. Although the measured ammonium concentrations remained within ranges generally considered physiological, the biological implications should thus be considered. However, as the current study showed protective effects on intestinal barrier function and immune responses, the increased ammonium concentrations would probably not contribute to any adverse health effect following repeated administration with the herbal test product.

Intestinal barrier dysfunction is present in many patients with IBS, especially those with the IBS-D subtype, where the proportion of patients with increased intestinal permeability ranges from 39% to 62% [46]. Butyrate is a key microbial metabolite that supports intestinal barrier function by acting as a critical energy source for colonocytes and increasing the expression of tight junction proteins [47,48]. In the present study, intestinal epithelial cells incubated with herbal test product-treated DC ferments had a significant increase in intestinal barrier integrity compared with the negative control, indicating a potential benefit for patients with IBS-D who may experience barrier dysfunction. This aligns with previous studies showing that two individual components of the herbal test product, myrrh and coffee charcoal, have a stabilizing effect on the intestinal barrier *in vitro* [8,11]. A potential mechanism for this is the ability of the test product to increase the abundance of butyrate-producing bacteria, such as *Butyricicoccus* and *Lachnospiraceae*, thereby increasing colonic butyrate levels which can support colonocyte health and tight junction protein expression. Indeed, it has been shown that addition of pure butyrate to the Caco-2/THP-1 co-culture system is able to protect Caco-2 cells and enhance TEER of the monolayer, while selectively increasing IL-6 and IL-10 secretion and inhibiting IL-1β and TNF-α secretion [49]. This observation strengthens the hypothesis that enhancement of microbiome activity, especially production of butyrate, could be linked to the observed increase in intestinal barrier integrity following administration of the herbal preparation.

There is some uncertainty around the role of inflammation in IBS; however, evidence suggests that low-level inflammation is likely involved [50]. The used Caco-2/THP-1 co-culture model in the current study reflects features observed in inflammatory conditions [31], with the differentiated Caco-2 cells displaying the characteristics of mature enterocyte-like cells, including tight junction formation and vectorial transport [51] and the phorbol-12-myristate-13-acetate-differentiated THP-1 cells resembling macrophage-like cells with features such as adhesion, migration and Toll-like receptor (TLR) responses [52]. Here, the herbal test product induced an anti-inflammatory response, with an increase in IL-10 in both the PC and DC. IL-6 levels were also increased following treatment. IL-6 can act as either a pro- or anti-inflammatory cytokine, depending on the microenvironment [53]. In the context of the present study, IL-6 is linked to protective effects on the intestinal barrier [54]. Interestingly, *Bacteroidales*, which was enriched with treatment, has been reported to recruit IL-6 producing intraepithelial lymphocytes to the colon to support barrier integrity [54]. This suggests the test products potential for improving the intestinal barrier in humans via *Bacteroidales* enrichment, though further investigation is required. Another compelling link between test product-induced changes in the microbiota composition and inflammation is the decreased abundance of *Veillonella* spp. with treatment. This is the only change that was observed consistently across both colon compartments and in both the luminal and mucosal environments, and it has been reported that an overabundance of *Veillonella parvula* is associated with intestinal inflammation [55]. Studies to further investigate this potential mechanism are of interest.

Several *in vitro* and animal model studies have demonstrated the anti-inflammatory effects of myrrh, chamomile extract, and coffee charcoal (reviewed in Vissiennon et al. [13]). For example, a study evaluating the effects of these individual compounds found that chamomile flower and coffee charcoal extracts resulted in a significant increase in IL-10 production by LPS-activated THP-1 cells [9]. In the same study, all three herbal compounds individually induced a dose-dependent decrease in TNF-α production. Another study found that coffee charcoal had a concentration-dependent anti-inflammatory effect, inhibiting TNF-α, IL-6, and monocyte chemoattractant protein (MCP)-1 release from LPS-activated THP-1 cells [10]. Chlorogenic acid isomers, particularly cryptochlorogenic acid, and caffeic acid were largely responsible for this inhibition. The combined herbal extracts (myrrh, chamomile, and coffee charcoal) demonstrated a synergistic effect in terms of their ability to reduce inflammation, highlighting the benefit of a mixed herbal formula [11]. The significant increase in IL-10 production observed in the present study is in agreement with these previous findings. Of note, the present study did not observe a decrease in TNF-α or IL-6 production by THP-1 cells in response to LPS stimulation. In fact, IL-6 levels were increased with the herbal test product. This is likely due to differences in experimental design, particularly the use of co-culture assays rather than THP-1 monocultures and the addition of colonic ferments in the co-culture assays which contain a complex mixture of microbial metabolites.

Importantly, this study was limited by the *in vitro* design and the number of patient samples. While the methods used allow for detailed mechanistic investigations that are not possible *in vivo*, the findings do not directly translate to the *in vivo* situation as the model (like any other *in vitro* gut model) lacks for instance the dynamic immune system, innervation, and systemic host feedback which could be highly relevant in the context of IBS-D. Even though the used *in vitro* design included evaluation of effects on gut barrier integrity and immune response by coupling samples generated from the M-SHIME^®^ system with co-culture cell assays, any findings must be further investigated and confirmed in clinical trials. Furthermore, while this study allowed for the assessment of some interindividual responses to product supplementation, the small number of donors limits the statistical power of the analysis. Indeed, with respect to microbial community diversity for instance, treatment effects did not surpass interindividual variability, thereby limiting the generalizability of the obtained results. Finally, the specific bioactive metabolite(s) responsible for the observed treatment effects were not further assessed during the current study, which could play a crucial role in understanding the mechanism of action of the herbal preparation in the area of gastrointestinal disorders. Further research could therefore be conducted to better understand which metabolites are of importance for driving potential host-microbiome interactions, for instance using in-depth metabolomics analyses.

## Conclusions

This study showed for the first time that the herbal combination of myrrh, chamomile extract, and coffee charcoal affected the gut microbiota of patients with IBS-D by stimulating increased SCFA production and enrichment of SCFA-producing bacterial families and genera in an *in vitro* model. Treated colonic ferments had a protective effect on barrier disruption and induced an anti-inflammatory response in co-culture models of intestinal inflammation. Together, these findings suggest that treatment with a herbal preparation of myrrh, chamomile extract, and coffee charcoal may impart beneficial effects on patients with IBS-D, which may potentially extend to other chronic gastrointestinal disorders. Further studies are needed to understand the effects of the herbal preparation (and the specific metabolites involved) on patients with IBS-D.

## Supporting information

S1 Fig Study design. DC, distal colon; PC, proximal colon; St/SI, stomach/small intestine.
(PDF)

S2 Fig Metabolic activity – lactate. Box plots showing changes in lactate levels (mM) between the negative control and treatment with the test product (myrrh, chamomile extract, and coffee charcoal) in the proximal and distal colon compartments across the treatment period (d1, d3, d5, d8) in M-SHIME^®^ colonic incubations. Results are presented in a box plot displaying the value for each of the four donors with a dot. The horizontal line represents the median across donors. Unpaired Student’s t-tests were used to compare changes observed for treatment with the test product versus the negative control. *p < 0.05. **p < 0.01. d, day; M-SHIME^®^, Mucosal Simulator of the Human Intestinal Microbial Environment; ns, not significant.
(PDF)

S3 Fig Beta-diversity. Hierarchical clustering to demonstrate beta-diversity in the luminal and mucosal environments of the PC and DC compartments of the M-SHIME^®^ colonic incubations on day 8 following treatment with the test product (myrrh, chamomile extract, and coffee charcoal) versus the negative control. DC, distal colon; LD, linear discriminant; M-SHIME^®^, Mucosal Simulator of the Human Intestinal Microbial Environment; PC, proximal colon.
(PDF)

S1 Text Processing and storage of fecal samples. (PDF)

S2 Text 16S-targeted sequencing, read assembly, and cleanup. (PDF)
