# Supplementary material for: Repeated dosing of myrrh, chamomile extract, and coffee charcoal reveals potential health-beneficial effects in patients with irritable bowel syndrome in the M-SHIME simulator
Source: PLoS One. 2026 May 27;21(5):e0348791. doi: 10.1371/journal.pone.0348791 (PMC13215480; doi:10.1371/journal.pone.0348791)

Repeated dosing of myrrh, chamomile extract, and coffee charcoal reveals potential health-beneficial effects in patients with irritable bowel syndrome in the M-SHIME® simulator

Meinolf Wonnemann et al.

Supporting information

S1 Fig. Study design. DC, distal colon; PC, proximal colon; St/SI, stomach/small intestine.

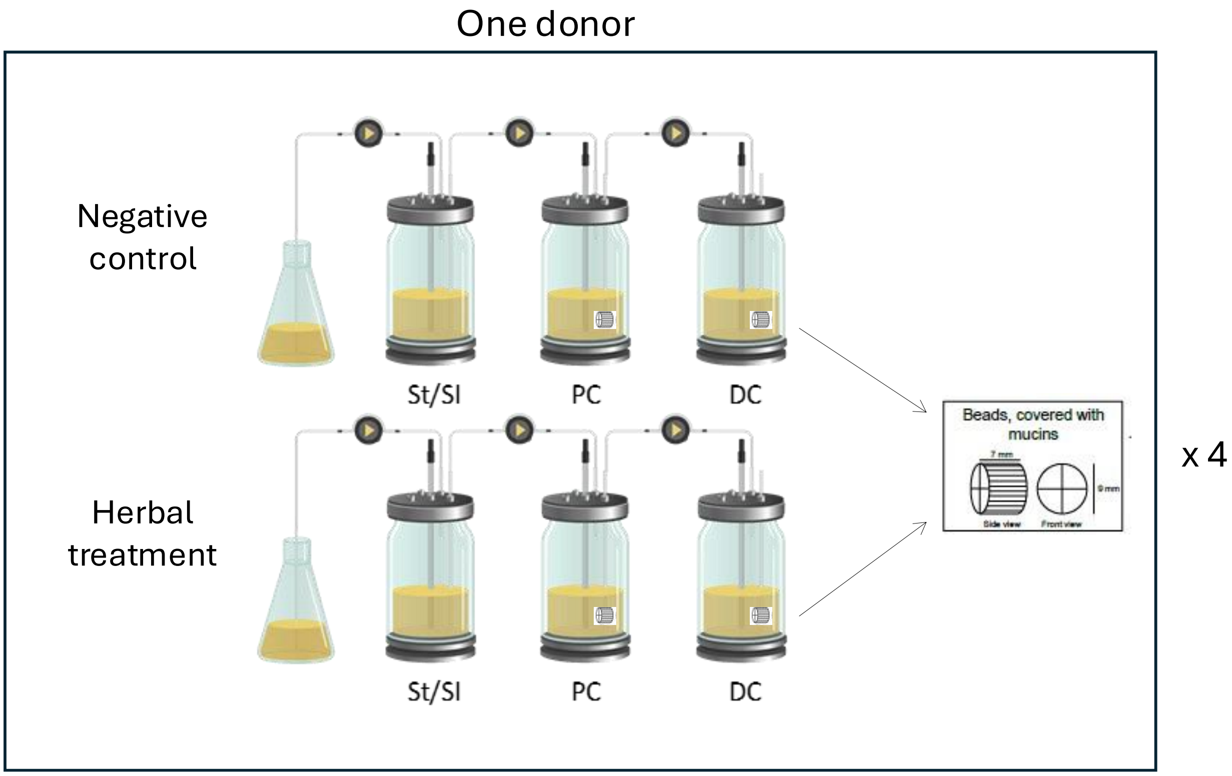

Supplement: S1 Fig — DC, distal colon; PC, proximal colon; St/SI, stomach/small intestine. (PDF) [file pone.0348791.s001.pdf]
