# Supplementary material for: Repeated dosing of myrrh, chamomile extract, and coffee charcoal reveals potential health-beneficial effects in patients with irritable bowel syndrome in the M-SHIME simulator
Source: PLoS One. 2026 May 27;21(5):e0348791. doi: 10.1371/journal.pone.0348791 (PMC13215480; doi:10.1371/journal.pone.0348791)

Repeated dosing of myrrh, chamomile extract, and coffee charcoal reveals potential health-beneficial effects in patients with irritable bowel syndrome in the M-SHIME® simulator

Meinolf Wonnemann et al.

Supporting information

S2 Fig. Metabolic activity - lactate. Box plots showing changes in lactate levels (mM) between the negative control and treatment with the test product (myrrh, chamomile extract, and coffee charcoal) in the proximal and distal colon compartments across the treatment period (d1, d3, d5, d8) in M-SHIME® colonic incubations. Results are presented in a box plot displaying the value for each of the four donors with a dot. The horizontal line represents the median across donors. Unpaired Student's t-tests were used to compare changes observed for treatment with the test product versus the negative control. *p<0.05. **p<0.01.

d, day; M-SHIME®, Mucosal Simulator of the Human Intestinal Microbial Environment; ns, not significant.

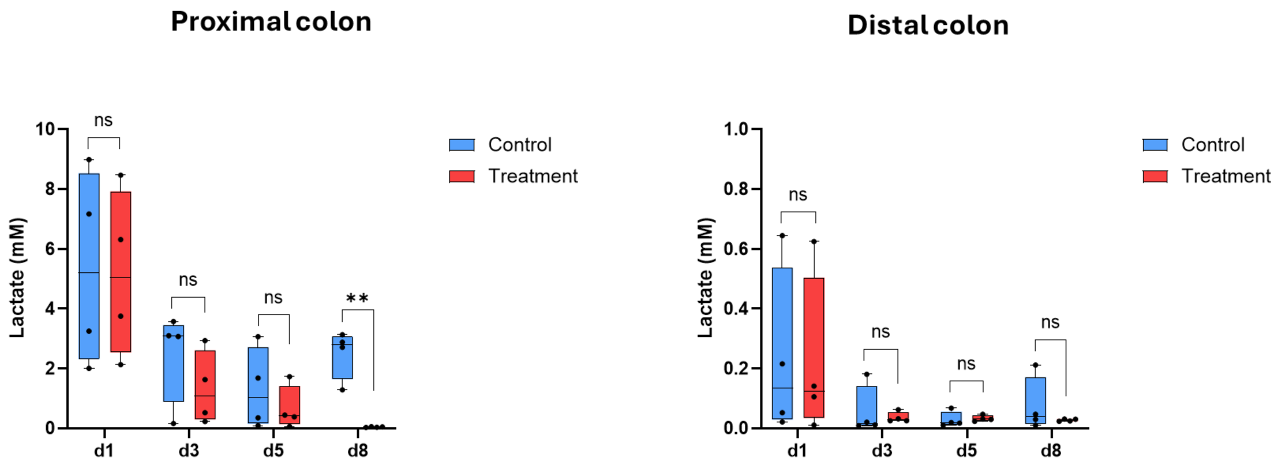

Supplement: S2 Fig — Box plots showing changes in lactate levels (mM) between the negative control and treatment with the test product (myrrh, chamomile extract, and coffee charcoal) in the proximal and distal colon compartments across the treatment period (d1, d3, d5, d8) in M-SHIME® colonic incubations. Results are presented in a box plot displaying the value for each of the four donors with a dot. The horizontal line represents the median across donors. Unpaired Student’s t-tests were used to compare changes observed for treatment with the test product versus the negative control. *p < 0.05. **p < 0.01. d, day; M-SHIME®, Mucosal Simulator of the Human Intestinal Microbial Environment; ns, not significant. (PDF) [file pone.0348791.s002.pdf]
