# Supplementary material for: Repeated dosing of myrrh, chamomile extract, and coffee charcoal reveals potential health-beneficial effects in patients with irritable bowel syndrome in the M-SHIME simulator
Source: PLoS One. 2026 May 27;21(5):e0348791. doi: 10.1371/journal.pone.0348791 (PMC13215480; doi:10.1371/journal.pone.0348791)

Repeated dosing of myrrh, chamomile extract, and coffee charcoal reveals potential health-beneficial effects in patients with irritable bowel syndrome in the M-SHIME® simulator

Meinolf Wonnemann et al.

Supporting information

S3 Fig. Beta-diversity. Hierarchical clustering to demonstrate beta-diversity in the luminal and mucosal environments of the PC and DC compartments of the M-SHIME® colonic incubations on day 8 following treatment with the test product (myrrh, chamomile extract, and coffee charcoal) versus the negative control.

DC, distal colon; LD, linear discriminant; M-SHIME®, Mucosal Simulator of the Human Intestinal Microbial Environment; PC, proximal colon.

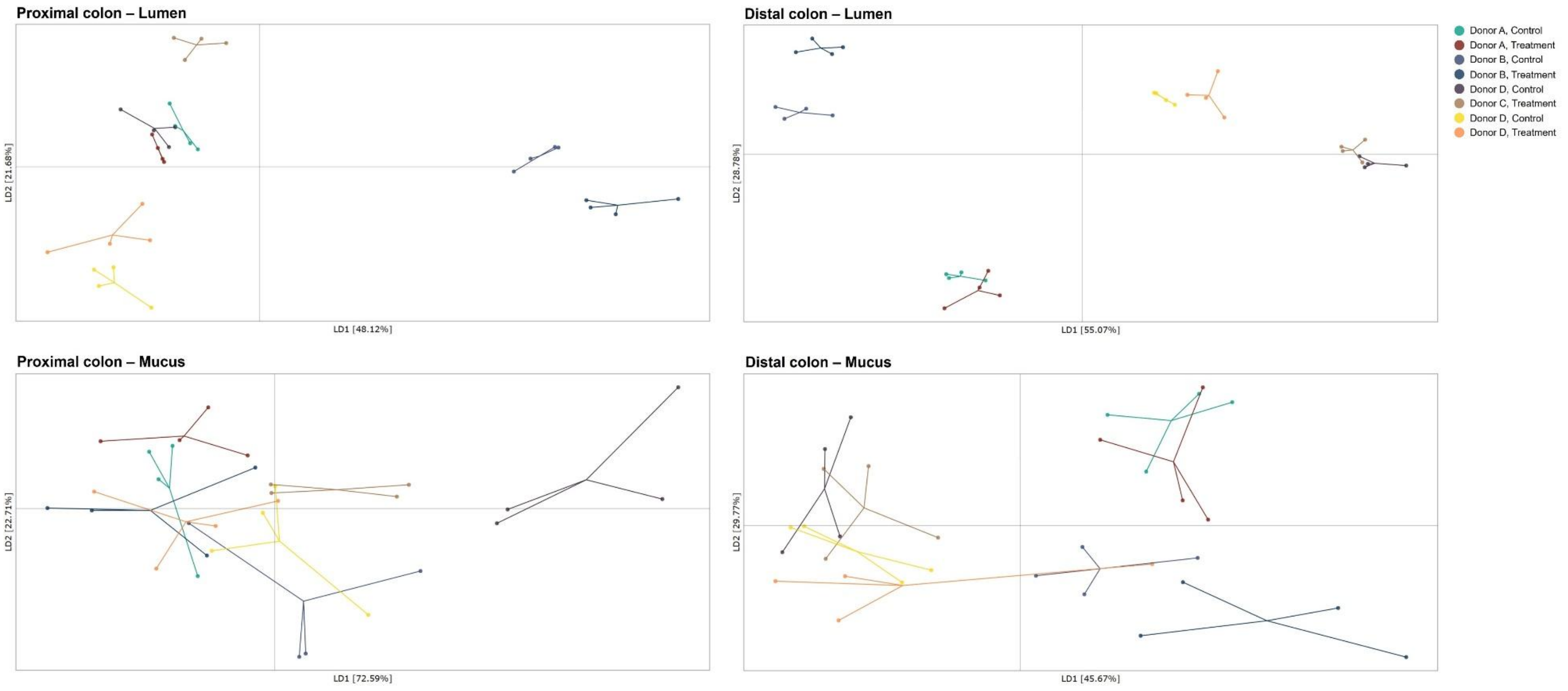

Supplement: S3 Fig — Hierarchical clustering to demonstrate beta-diversity in the luminal and mucosal environments of the PC and DC compartments of the M-SHIME® colonic incubations on day 8 following treatment with the test product (myrrh, chamomile extract, and coffee charcoal) versus the negative control. DC, distal colon; LD, linear discriminant; M-SHIME®, Mucosal Simulator of the Human Intestinal Microbial Environment; PC, proximal colon. (PDF) [file pone.0348791.s003.pdf]
