# Supplementary material for: Repeated dosing of myrrh, chamomile extract, and coffee charcoal reveals potential health-beneficial effects in patients with irritable bowel syndrome in the M-SHIME simulator
Source: PLoS One. 2026 May 27;21(5):e0348791. doi: 10.1371/journal.pone.0348791 (PMC13215480; doi:10.1371/journal.pone.0348791)
Supplement: S1 Text — (PDF) [file pone.0348791.s004.pdf]

Repeated dosing of myrrh, chamomile extract, and coffee charcoal reveals potential health-beneficial effects in patients with irritable bowel syndrome in the M-SHIME® simulator

Meinolf Wonnemann et al.

Supporting information

S1 Text. Processing and storage of fecal samples.

After collection, fecal samples were homogenized with phosphate buffered saline in an anaerobic environment. The samples were briefly centrifuged to remove large particles, an equal volume of optimized in-house cryoprotectant (modified from Hoefman et al. [1]) was added, and the suspensions were flash frozen in liquid nitrogen and stored at –80°C. At the start of the *in vitro* simulations, samples were defrosted and immediately added to the M-SHIME® colonic reactors.

References

1. Hoefman S, Pommerening-Roser A, Samyn E, et al. Efficient cryopreservation protocol enables accessibility of a broad range of ammonia-oxidizing bacteria for the scientific community. *Res Microbiol* 2013;164:288-92.
