# Supplementary material for: Repeated dosing of myrrh, chamomile extract, and coffee charcoal reveals potential health-beneficial effects in patients with irritable bowel syndrome in the M-SHIME simulator
Source: PLoS One. 2026 May 27;21(5):e0348791. doi: 10.1371/journal.pone.0348791 (PMC13215480; doi:10.1371/journal.pone.0348791)
Supplement: S2 Text — (PDF) [file pone.0348791.s005.pdf]

Repeated dosing of myrrh, chamomile extract, and coffee charcoal reveals potential health-beneficial effects in patients with irritable bowel syndrome in the M-SHIME® simulator

Meinolf Wonnemann et al.

Supporting information

S2 Text. 16S-targeted sequencing, read assembly, and cleanup.

16S-targeted sequencing was accomplished using primers spanning two hypervariable regions (V3-V4) of the 16S rRNA gene, 341F, 5′-CCTACGGGNGGCWGCAG-3′ and 785R, 5′-GACTACHVGGGTATCTAAKCC-3′) [1,2]. Pair-end sequencing of 2 × 250 bp yielded 424 bp amplicons (LGC Genomics GmbH, Berlin, Germany). The MiSeq protocol from the Schloss lab was used for read assembly and cleanup [2,3]. Briefly, reads were assembled into contigs, alignment-based quality filtering was performed (alignment to the mothur-reconstructed SILVA SEED alignment, v138), chimeras were removed (vsearch v2.13.3), naïve Bayesian classifier [4] and SILVA NR v138_1 were employed to assign taxonomy, and mothur (v.1.44.3) was used to cluster contigs into Operational Taxonomic Units (OTUs) at 97% sequence similarity. Sequences were removed if they were classified as Eukaryota, Archaea, chloroplasts, or mitochondria, or could not be classified. Each OTU was represented by its most abundant sequence.

References

1. Klindworth A, Pruesse E, Schweer T, et al. Evaluation of general 16S ribosomal RNA gene PCR primers for classical and next-generation sequencing-based diversity studies. *Nucleic Acids Res* 2013;41:e1.
2. Kozich JJ, Westcott SL, Baxter NT, et al. Development of a dual-index sequencing strategy and curation pipeline for analyzing amplicon sequence data on the MiSeq Illumina sequencing platform. *Appl Environ Microbiol* 2013;79:5112-20.
3. Schloss PD, Westcott SL. Assessing and improving methods used in operational taxonomic unit-based approaches for 16S rRNA gene sequence analysis. *Appl Environ Microbiol* 2011;77:3219-26.
4. Wang Q, Garrity GM, Tiedje JM, et al. Naive Bayesian classifier for rapid assignment of rRNA sequences into the new bacterial taxonomy. *Appl Environ Microbiol* 2007;73:5261-7.
